# Polyarteritis nodosa and antiphospholipid syndrome: a systematic review of a rare and challenging overlap between vasculitis and thrombotic vasculopathy

**DOI:** 10.1007/s10067-026-08214-8

**Published:** 2026-06-30

**Authors:** Jozélio Freire de Carvalho, Roberto Paulo Correia de Araujo

**Affiliations:** Instituto de Ciências da Saúde, Universidade Federal da Bahia, Av. Reitor Miguel Calmon, s/n-Canela, Salvador, BA 40231-300 Brazil

**Keywords:** Antiphospholipid syndrome, Autoimmunity, Medium-vessel vasculitis, Polyarteritis nodosa, Thrombosis, Vasculitis

## Abstract

**Background:**

The coexistence of polyarteritis nodosa (PAN) and antiphospholipid syndrome (APS) is rarely reported and remains poorly characterized. Both conditions may involve vascular injury, but through distinct mechanisms—necrotizing inflammation in PAN and thrombotic vasculopathy in APS. Their overlap may result in a unique and potentially severe clinical phenotype, posing diagnostic and therapeutic challenges. In addition, differentiating inflammatory vasculitic injury from thrombotic vascular occlusion may be particularly difficult in this overlap setting, especially in patients with catastrophic presentations.

**Objective:**

To systematically review all published cases describing the association between PAN and APS, focusing on clinical features, laboratory findings, pathophysiological insights, management strategies, and outcomes.

**Methods:**

A systematic search was conducted in PubMed/MEDLINE, Scopus, and Web of Science from inception through 2026. The search included terms related to “polyarteritis nodosa” and “antiphospholipid syndrome.” Only studies reporting cases fulfilling accepted diagnostic or classification criteria for PAN and APS were included. Data extraction encompassed demographic characteristics, temporal relationship between diseases, clinical manifestations, antiphospholipid antibody profiles, imaging and histopathological findings, treatments, and outcomes. Due to heterogeneity and limited sample size, a descriptive analysis was performed. Because of the rarity of this overlap syndrome, the available evidence consisted predominantly of case reports and small case series, which were critically analyzed within a systematic review framework.

**Results:**

A small number of cases were identified, all reported as case reports or small case series. Most patients were middle-aged males, although one pediatric case was described. The temporal relationship varied, with simultaneous diagnosis being most common, while in some cases APS developed after PAN onset. Clinically, patients exhibited features of systemic PAN, including visceral involvement, neuropathy, and hypertension, alongside thrombotic events characteristic of APS, such as arterial occlusions, renal infarctions, stroke, and limb ischemia. Antiphospholipid antibodies—particularly anticardiolipin antibodies and lupus anticoagulant—were frequently detected. A distinctive feature was the coexistence of aneurysmal lesions and thrombosis. Treatment generally included corticosteroids and cyclophosphamide, often combined with anticoagulation. Outcomes were heterogeneous, ranging from clinical improvement to severe complications, including amputations and death. Several reports also raised the possibility of catastrophic antiphospholipid syndrome (CAPS), further complicating the distinction between inflammatory multiorgan vasculitis and thrombotic microangiopathic disease.

**Conclusions:**

The association between PAN and APS represents a rare but clinically significant overlap syndrome characterized by the interplay between inflammatory vasculitis and thrombotic mechanisms. Recognition of this entity is essential, as it has important implications for diagnosis and management. Emerging evidence suggests that endothelial dysfunction, complement activation, NETosis, and thromboinflammatory pathways may contribute to the pathogenesis of this overlap phenotype. Future studies are needed to better define its pathogenesis, optimize therapeutic strategies, and improve outcomes.

## Introduction

Polyarteritis nodosa (PAN) is a necrotizing vasculitis of medium-sized arteries characterized by transmural inflammation and fibrinoid necrosis, with a tendency toward aneurysm formation and organ ischemia [1]. Clinically, PAN presents with heterogeneous manifestations, including constitutional symptoms, hypertension, renal infarction, peripheral neuropathy, and gastrointestinal involvement, reflecting its multisystemic vascular distribution [1, 2].

Antiphospholipid syndrome (APS), in contrast, is an autoimmune thrombotic disorder characterized by recurrent arterial and/or venous thrombosis and/or pregnancy morbidity in association with persistent antiphospholipid antibodies (aPL), including anticardiolipin antibodies, anti-β2 glycoprotein I, and lupus anticoagulant [3]. APS is classically defined as a thrombotic vasculopathy rather than a primary inflammatory vasculitis; however, growing evidence supports the involvement of complement activation, endothelial dysfunction, and inflammatory pathways in its pathogenesis [4, 5]. Recent studies have further demonstrated the involvement of neutrophil extracellular traps (NETosis), endothelial microparticles, complement-mediated vascular injury, and endothelial microRNA dysregulation in APS pathogenesis, reinforcing the concept of APS as a thromboinflammatory disorder rather than a purely thrombotic condition [4, 5]. Interestingly, several of these mechanisms overlap with pathways implicated in vascular inflammation and endothelial injury in systemic vasculitides, raising the possibility of shared pathogenic networks between APS and PAN.

Distinguishing vasculitis from thrombosis is not merely semantic but has important therapeutic implications. While PAN is typically managed with aggressive immunosuppression, APS requires long-term anticoagulation. The coexistence of both conditions introduces significant diagnostic and therapeutic complexity, particularly because vascular lesions may result from either inflammatory destruction or thrombotic occlusion [6].

Although APS has been described in association with both small- and large-vessel vasculitides, its relationship with PAN remains poorly defined [6, 7]. The available literature is largely limited to isolated case reports and small case series describing patients with clinical, angiographic, or histopathological features consistent with PAN in the presence of definite or probable APS [8–11]. In these cases, overlapping manifestations—such as arterial aneurysms, infarctions, and livedoid or necrotic skin lesions—often obscure the distinction between true necrotizing vasculitis and severe thrombotic vasculopathy. This distinction becomes even more challenging in patients presenting with catastrophic clinical manifestations involving multiple organs, in whom catastrophic antiphospholipid syndrome (CAPS) may clinically mimic severe systemic vasculitis. In such scenarios, differentiating inflammatory vascular destruction from diffuse thrombotic microvascular injury has direct implications for immunosuppressive and anticoagulant management.

Furthermore, antiphospholipid antibodies have been identified in a subset of patients with primary systemic vasculitides [2], raising uncertainty as to whether these antibodies represent non-specific epiphenomena, direct contributors to vascular injury, or markers of a distinct overlap phenotype [7]. This issue is particularly relevant in PAN, where characteristic vascular imaging findings, such as microaneurysms, may coexist with thrombotic events, complicating etiological interpretation.

To date, no systematic review has specifically addressed the association between PAN and APS. Given the rarity of this overlap and its potential impact on clinical management, a systematic synthesis of the available evidence is warranted.

This study aims to systematically analyze all reported cases of coexisting PAN and APS, focusing on clinical presentation, laboratory findings, imaging, histopathology, therapeutic strategies, and outcomes. Beyond a descriptive synthesis, particular emphasis was placed on diagnostic dilemmas, thromboinflammatory mechanisms, the potential overlap with catastrophic APS, and therapeutic challenges related to anticoagulation in the presence of aneurysmal disease. By doing so, we seek to better characterize this rare overlap and provide insights into its diagnostic and therapeutic implications.

## Methods

This systematic review was conducted in accordance with the Preferred Reporting Items for Systematic Reviews and Meta-Analyses (PRISMA 2020) guidelines [12].

## Study design and research question

We conducted a systematic review to identify and synthesize all published evidence describing the coexistence of PAN and APS. The primary objective was to characterize the clinical presentation, laboratory findings, imaging and histopathological features, therapeutic approaches, and outcomes associated with this rare overlap. The research question guiding this review was focused on determining the clinical characteristics, diagnostic patterns, management strategies, and outcomes of patients with coexisting PAN and APS (Tables 1 and 2).

**Table 1:** Clinical characteristics of reported cases of PAN associated with APS

Author, year (Ref)	Age/sex	Temporal relationship	PAN involvement	APS manifestations	aPL profile	Vascular pattern	Treatment	Outcome
Schoonjans 1996 [1]	52/M	Simultaneous	Visceral PAN, neuropathy	Recurrent thrombosis	aCL ↑	Mixed (vasculitis + thrombosis)	Steroids + cyclophosphamide	Improvement
Dasgupta 1997 [2]	64/F	Simultaneous	Renal PAN, aneurysms	Iliofemoral thrombosis	LA +, aCL ↑	Aneurysm + thrombosis	Immunosuppression ± anticoagulation	Improvement
Morelli 1998 [3]	42/F	APS after PAN	Systemic PAN	Stroke, valvular disease	aCL ↑, LA +	Thrombosis predominant	Steroids + anticoagulation	Death
Han 2004 [4]	44/M	Simultaneous	Severe systemic PAN	Catastrophic APS	aCL ↑	Multiorgan thrombosis	Steroids + cyclophosphamide	Death
Costa 2006 [5]	37/M	Simultaneous	Severe PAN, aneurysms	Arterial thrombosis	aCL ↑	Mixed (aneurysm + thrombosis)	Immunosuppression + anticoagulation	Severe
Musuruana 2008 [6]	43/M	APS after PAN	Chronic PAN	Limb ischemia, myocardial infarction	aCL ↑, LA +	Thrombosis without inflammation	Steroids + cyclophosphamide + anticoagulation	Amputation, death
Huang 2009 [7]	34/M	Simultaneous	Renal PAN	Renal infarction	LA +	Aneurysm + infarction	Steroids + cyclophosphamide + anticoagulation	Recovery
Valeyrie 2003 [8]	Adult	Simultaneous	Systemic PAN	CNS ischemia	aPL +	Thrombotic CNS involvement	Immunosuppression + anticoagulation	Variable
Caldas 2013 [9]	Adult	Simultaneous	PAN (aneurysm, neuropathy)	Stroke, thrombosis	aCL ↑/LA +	Mixed pattern	Immunosuppression + anticoagulation	Improvement
Nimbvikar 2026 [10]	Adult	Overlap/evolving	Cutaneous and systemic PAN	Catastrophic APS features	Persistent aPL	Mixed (vasculitis + thrombosis)	Steroids + anticoagulation	Improvement
Yildiz 2013 [11]	16/M	Simultaneous	Renal PAN, neuropathy	Probable catastrophic APS	aCL ↑, LA +	Renal thrombosis + infarction	Steroids + cyclophosphamide + LMWH	Improvement

*PAN* polyarteritis nodosa, *APS* antiphospholipid syndrome, *aPL* antiphospholipid antibodies, *aCL* anticardiolipin antibodies, *LA* lupus anticoagulant, *CNS* central nervous system, *LMWH* low-molecular-weight heparin

**Table 2:** Comparative features between isolated PAN and PAN associated with APS

Feature	Isolated PAN	PAN associated with APS
Pathophysiology	Necrotizing inflammation of medium-sized arteries	Combined mechanism: necrotizing vasculitis plus thrombotic vasculopathy mediated by antiphospholipid antibodies
Vascular involvement	Predominantly inflammatory arterial lesions	Coexistence of inflammatory lesions and thrombotic occlusions
Aneurysms	Frequent, especially in renal and mesenteric arteries	Frequent, often coexisting with thrombosis and infarction
Thrombosis	Uncommon and usually secondary to inflammation	Central feature, including arterial and occasionally venous thrombosis
Histopathology	Fibrinoid necrosis with inflammatory infiltrates in vessel wall	Mixed pattern: inflammatory vasculitis and/or thrombosis with minimal inflammation
Antiphospholipid antibodies	Typically absent	Present (anticardiolipin, lupus anticoagulant, anti-β2 glycoprotein I)
Renal involvement	Hypertension, ischemia due to vasculitis	Renal infarction, renovascular hypertension, thrombosis of renal arteries
Neurological involvement	Mononeuritis multiplex, less commonly stroke	Increased frequency of ischemic stroke and CNS thrombosis
Peripheral ischemia	Possible but less severe	More frequent and severe, including digital necrosis and limb ischemia
Cardiac involvement	Hypertension-related or ischemic complications	May include APS-related valvular disease and myocardial infarction
Clinical course	Variable, often responsive to immunosuppression	Often more severe, with higher morbidity and risk of recurrence
Diagnostic challenge	Relatively well-defined with angiography and biopsy	Significant overlap with APS manifestations; differentiation between vasculitis and thrombosis may be difficult
Imaging findings	Microaneurysms and segmental stenosis	Combination of microaneurysms, arterial occlusion, and infarction
Treatment approach	Corticosteroids and immunosuppressive agents (e.g., cyclophosphamide)	Combination therapy: immunosuppression plus anticoagulation, requiring individualized risk–benefit assessment
Therapeutic dilemma	Mainly control of inflammation	Balance between anticoagulation (thrombosis prevention) and bleeding risk due to aneurysms
Prognosis	Generally favorable with appropriate treatment	More heterogeneous; increased risk of severe complications, including amputations and death

Although the available literature was expected to consist predominantly of case reports and small case series due to the rarity of this overlap syndrome, a systematic review methodology was rigorously applied, including predefined eligibility criteria, structured database searches, independent screening, and standardized data extraction according to PRISMA 2020 recommendations.

## Search strategy

A comprehensive literature search was performed in PubMed/MEDLINE, Scopus, and Web of Science from database inception through March 2026. The search strategy combined Medical Subject Headings and free-text terms related to PAN and APS, including “polyarteritis nodosa,” “antiphospholipid syndrome,” “antiphospholipid antibodies,” “lupus anticoagulant,” “anticardiolipin,” and “anti-beta 2 glycoprotein I.” The search syntax was adapted for each database as appropriate. No restrictions were applied regarding language, publication date, or patient age. In addition, the reference lists of all included studies and relevant reviews were manually screened to identify additional eligible publications.

## Eligibility criteria

Studies were considered eligible if they reported individual cases or case series of patients with a diagnosis of PAN based on clinical, angiographic, or histopathological criteria consistent with established definitions, in association with APS defined by accepted classification criteria or clearly compatible clinical and laboratory features. Cases presenting catastrophic or multiorgan thrombotic manifestations compatible with CAPS were also considered when sufficient clinical evidence supported concomitant PAN-related vasculitic involvement. Only studies providing sufficient clinical detail to confirm the coexistence of both conditions were included. Original articles, including case reports, case series, and observational studies, were eligible for inclusion.

Studies were excluded if they reported isolated positivity of antiphospholipid antibodies without fulfilling clinical criteria for APS, if the diagnosis of PAN was uncertain or better explained by another vasculitis or non-inflammatory vasculopathy, or if they consisted of reviews, editorials, or expert opinions without original patient data. Studies with unavailable full text and insufficient extractable data were also excluded.

## Study selection

All records identified through the database search were imported into a reference management system, and duplicates were removed. Titles and abstracts were independently screened for eligibility, followed by full-text review of potentially relevant studies. Disagreements were resolved through discussion and consensus. The study selection process was documented according to PRISMA 2020 recommendations.

## Data extraction

Data were extracted using a standardized approach, focusing on demographic characteristics, type of PAN, clinical manifestations, APS-related events, antiphospholipid antibody profile, imaging findings, histopathological features, treatment strategies, and clinical outcomes. Special attention was given to the distinction between inflammatory vasculitic manifestations and thrombotic vascular injury, including possible catastrophic APS presentations, aneurysmal disease, and ischemic complications.

## Quality assessment

Given the expected predominance of case reports and case series, methodological quality was assessed using an adapted version of the Joanna Briggs Institute critical appraisal tools for case-based evidence. The assessment focused on clarity of diagnosis, completeness of clinical description, and adequacy of outcome reporting.

## Data synthesis

Due to the rarity of the condition and the anticipated heterogeneity of the available data, a qualitative synthesis was performed. The analysis emphasized clinical patterns, diagnostic challenges, therapeutic strategies, and outcomes, with additional critical evaluation of thromboinflammatory mechanisms, catastrophic APS overlap, anticoagulation dilemmas in aneurysmal disease, and the potential pathogenic contribution of antiphospholipid antibodies in vascular injury.

## Ethics statement

As this study was based exclusively on previously published data, ethical approval and informed consent were not required.

## Results

**Fig. 1:**
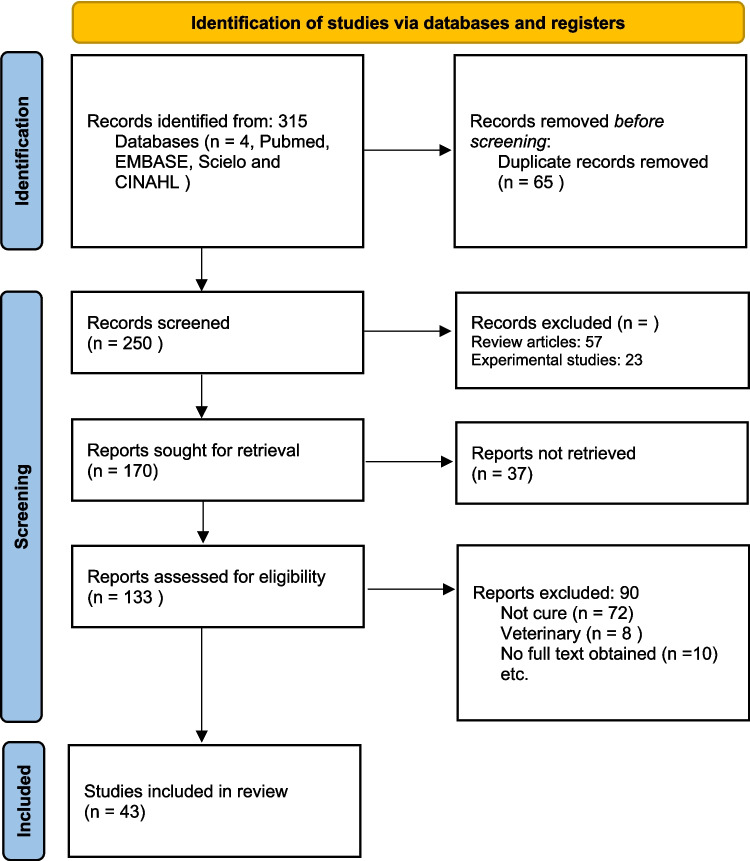
PRISMA flow diagram of study selection for the systematic review of PAN associated with APS

A systematic literature search identified a limited number of eligible studies reporting the coexistence of PAN and APS, all of which consisted of case reports or small case series [1–11]. The absence of cohort studies or controlled investigations highlights the lack of high-level evidence. Overall, fewer than a dozen well-documented cases were identified, underscoring the rarity of this overlap. The study selection process is summarized in a PRISMA flow diagram, detailing the number of records identified, screened, excluded, and included in the qualitative synthesis (Fig. 1).

The demographic profile showed a predominance of male patients, with ages ranging from adolescence to late adulthood. Most cases occurred in middle-aged individuals, although one pediatric case was reported [9]. The temporal relationship between PAN and APS was variable. In most reports, both conditions were diagnosed simultaneously [1, 2, 5, 7, 9], whereas in others APS developed years after the diagnosis of PAN, suggesting that antiphospholipid autoimmunity may emerge during the disease course rather than being present at baseline [3, 6].

Clinically, all patients presented with features consistent with systemic PAN, including constitutional symptoms, neuropathy, hypertension, and multiorgan involvement. Visceral arterial involvement was particularly prominent, especially affecting renal, mesenteric, and peripheral vascular beds [1, 2, 7]. Angiographic findings commonly demonstrated microaneurysms and segmental arterial stenosis, which are characteristic of PAN. However, in contrast to isolated PAN, these cases frequently exhibited concomitant thrombotic events, including arterial occlusions, renal infarctions, cerebrovascular events, and critical limb ischemia [3, 6, 7, 9].

Antiphospholipid antibodies were consistently detected, most commonly anticardiolipin antibodies and lupus anticoagulant [1, 6, 9]. These antibodies were often present at moderate to high titers and, in several cases, persisted over time, fulfilling classification criteria for APS. Notably, some patients developed thrombotic events in the absence of active inflammatory vascular lesions on histopathology, suggesting a dissociation between vasculitic and thrombotic mechanisms [6].

Renal involvement was among the most frequently reported manifestations, typically presenting as renovascular hypertension and renal infarctions [2, 7, 9]. Central nervous system involvement, including ischemic stroke, was also common and often associated with worse outcomes [3, 4]. In more severe cases, peripheral ischemia progressed to digital necrosis or limb loss, reflecting the combined effects of vascular inflammation and thrombosis [6].

**Fig. 2:**
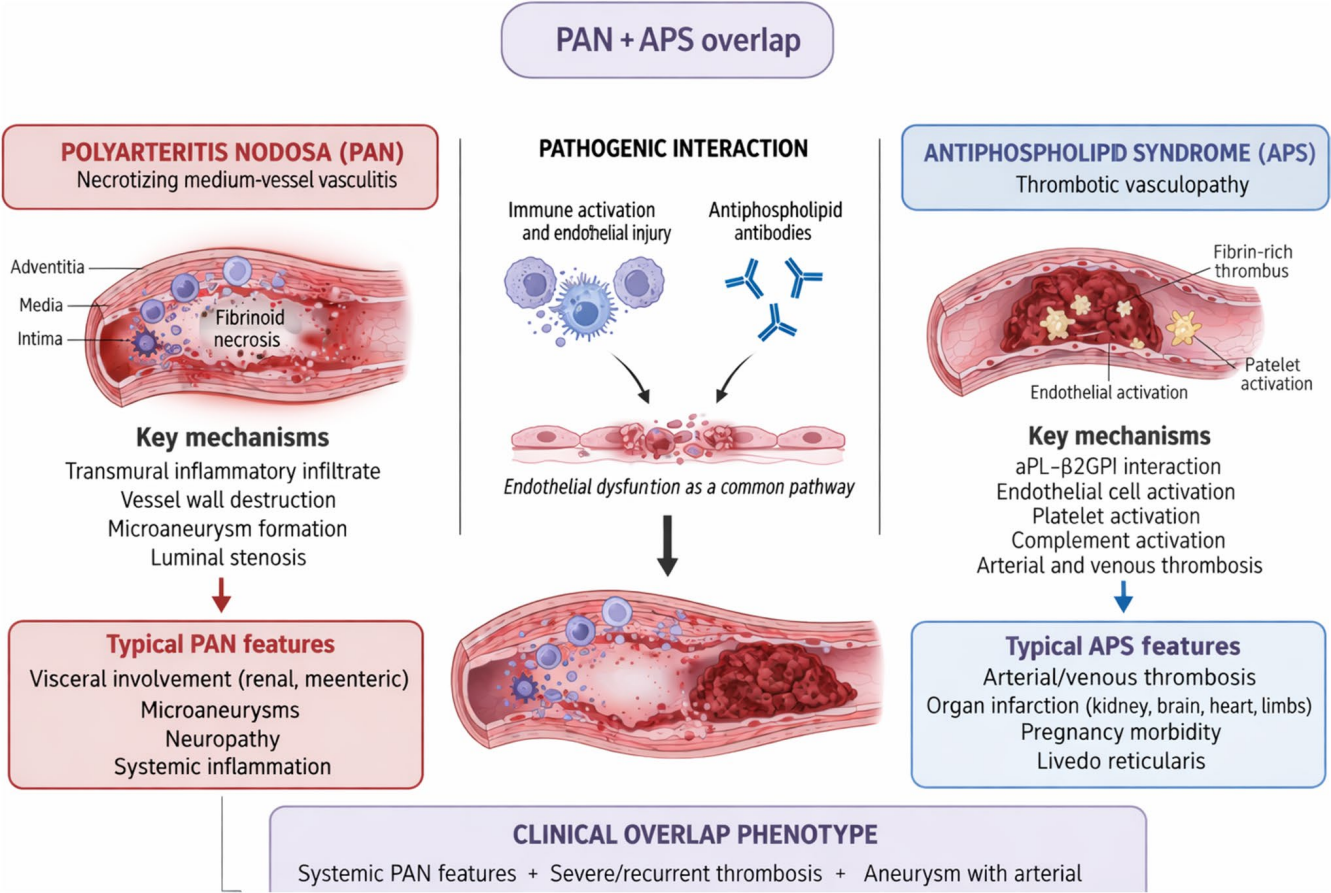
Integrated pathophysiological model of vascular injury in PAN associated with APS. This figure illustrates the integrated mechanisms underlying vascular injury in the overlap between PAN and APS. PAN is characterized by necrotizing inflammation of medium-sized arteries, leading to transmural inflammatory infiltrates, fibrinoid necrosis, and microaneurysm formation. In parallel, APS is driven by antiphospholipid antibodies, which induce endothelial activation, platelet aggregation, complement activation, NETosis, endothelial microparticle release, and thromboinflammatory signaling and thrombus formation. A central unifying mechanism is endothelial dysfunction, resulting from both immune-mediated vascular inflammation and antibody-mediated endothelial activation. This interaction creates a self-amplifying loop that promotes simultaneous vasculitic damage and superimposed thrombosis within the same vascular territory. The overlap phenotype is therefore defined by combined vascular injury, in which inflammatory arterial lesions coexist with thrombotic occlusions, leading to organ ischemia and infarction. Clinically, this dual process manifests as a more severe and complex phenotype, characterized by recurrent thrombosis, aneurysm formation with risk of rupture, and involvement of multiple organ systems, including kidneys, central nervous system, heart, and peripheral circulation. These mechanisms have direct therapeutic implications, as optimal management requires a balanced approach combining immunosuppressive therapy to control vasculitis and anticoagulation to prevent thrombotic events, while carefully considering the potential bleeding risk associated with aneurysmal disease

The association between PAN and APS can be conceptualized as a dual-hit model of vascular injury, in which inflammatory and thrombotic mechanisms coexist and interact. Necrotizing vasculitis in PAN leads to structural damage of medium-sized arteries, including fibrinoid necrosis and microaneurysm formation. Concurrently, antiphospholipid antibodies promote endothelial activation, platelet aggregation, and complement-mediated amplification of coagulation pathways, resulting in a prothrombotic state. The convergence of these processes results in endothelial dysfunction as a central pathogenic event, ultimately leading to combined vascular injury characterized by inflammation-induced damage with superimposed thrombosis. This integrated model helps explain the increased severity, diagnostic complexity, and broader clinical spectrum observed in patients with overlapping disease (Fig. 2). Emerging thromboinflammatory mechanisms potentially involved in this overlap include complement activation, NETosis, endothelial microparticles, and endothelial dysfunction associated with antiphospholipid antibodies.

A distinctive pathophysiological feature of APS-associated PAN is the coexistence of aneurysmal disease and thrombosis. This biphasic vascular phenotype complicates both diagnosis and management, as anticoagulation may increase the risk of aneurysm rupture, while immunosuppression alone may be insufficient to control thrombotic risk [7]. The role of antiphospholipid antibodies remains uncertain: some authors consider them an epiphenomenon secondary to endothelial injury, whereas others propose a direct pathogenic role in thrombosis [9].

Therapeutically, most patients received high-dose corticosteroids in combination with cyclophosphamide, reflecting standard management for severe PAN [1, 6, 7]. Anticoagulation was frequently added, particularly in patients with confirmed APS. Despite aggressive treatment, outcomes were heterogeneous. While some patients showed clinical improvement or stabilization [1, 7, 9], others developed severe complications, including amputations and death, particularly in cases with catastrophic APS or extensive vascular involvement [4, 6]. In some reports, distinguishing CAPS from severe multisystem PAN proved particularly challenging, especially in patients presenting with diffuse ischemic manifestations and multiorgan dysfunction.

## Discussion

The present review reinforces that the coexistence of PAN and APS represents a rare but clinically meaningful overlap, marked by substantial diagnostic uncertainty and relevant therapeutic consequences [1–10]. The available evidence is almost entirely restricted to case reports and small case series, which limits robust inference but, at the same time, reveals a remarkably consistent clinical pattern: patients frequently present with manifestations attributable both to inflammatory medium-vessel vasculitis and to thrombotic vasculopathy. This duality appears to be the central pathobiological and clinical hallmark of the PAN–APS association.

A first important point is that the association seems to produce a vascular phenotype more severe than would be expected from either condition alone. In the cases reviewed, aneurysms, stenoses, arterial occlusions, renal infarctions, cerebrovascular ischemia, digital necrosis, and limb-threatening ischemia often coexisted in the same patient [1, 2, 4–10]. Classical PAN is, by definition, a necrotizing vasculitis of medium-sized arteries, and microaneurysms on angiography remain one of its most recognizable features. APS, in contrast, is fundamentally a thrombotic vasculopathy. When these two mechanisms coexist, vascular injury may be amplified by the combination of endothelial inflammation, vessel wall destruction, luminal narrowing, and superimposed thrombosis. This may explain why several of the reported cases evolved with particularly aggressive ischemic complications, including stroke, myocardial infarction, bilateral renal infarction, and amputations [2, 4, 6–10].

A second major issue concerns diagnosis. In clinical practice, differentiating true necrotizing vasculitis from thrombosis-driven vascular occlusion can be extremely difficult. This challenge is especially evident when patients present with livedo reticularis, digital ischemia, neurologic events, renal dysfunction, or skin ulceration, since all of these may occur in either PAN or APS [2, 7, 9, 10]. The distinction becomes even more difficult in catastrophic clinical scenarios involving multiorgan dysfunction, where CAPS may clinically mimic fulminant systemic vasculitis. In such cases, diffuse thrombotic microvascular injury and inflammatory vascular destruction may coexist, making clinicopathological correlation essential for therapeutic decision-making. The recent 2026 case-based review by Nimbvikar et al. illustrates this problem particularly well: the patient initially carried a diagnosis of catastrophic APS, but persistent symptoms despite anticoagulation, together with skin biopsy evidence of medium-vessel vasculitis, later supported concomitant PAN [10]. Likewise, Caldas and de Carvalho emphasized that livedo, seizures, and stroke may belong to the spectrum of both disorders, and that the presence of angiographic microaneurysms may be especially helpful when PAN is suspected in a patient who also fulfills APS criteria [9].

Histopathology and angiography remain central in this distinction, but neither is infallible. In several cases, angiography documented classic PAN-type lesions, including renal and visceral microaneurysms, while biopsy showed either necrotizing vasculitis, thrombosis, or both [1, 2, 5, 6, 9, 10]. This mixed histopathological picture is highly informative. In some patients, inflammatory vessel wall destruction predominated; in others, thrombi were seen with little or no active inflammatory infiltrate, suggesting that APS may at times become the dominant process during the course of otherwise established PAN [6]. The case reported by Musuruana and Cavallasca is particularly illustrative in this regard: years after the initial diagnosis of PAN, the patient developed progressive ischemic lesions and histologic thrombosis without inflammatory infiltrates, supporting the view that APS may emerge later and substantially modify the phenotype and prognosis of PAN [6].

The temporal relationship between the two disorders deserves attention. Although many patients seem to be diagnosed with PAN and APS simultaneously, the literature also shows that APS may appear years after the onset of vasculitis [3, 6, 9]. This observation suggests that the coexistence is not always merely coincidental at presentation. One possibility is that chronic vascular inflammation and endothelial injury in PAN expose phospholipid-related or endothelial antigens, thereby favoring the appearance of antiphospholipid antibodies in predisposed individuals. This mechanism has been speculated by previous authors and is explicitly discussed by Caldas and de Carvalho, who note that endothelial disruption in vasculitis may generate immunologically hidden antigens and stimulate anti-endothelial and antiphospholipid responses [9]. Another, more conservative interpretation is that antiphospholipid antibodies may occasionally represent an epiphenomenon of intense inflammation or polyclonal immune activation, without necessarily being the main driver of vascular thrombosis in every patient. Current evidence is insufficient to resolve this question definitively.

This uncertainty is mirrored in the broader vasculitis literature. Rees et al., as cited in both Caldas et al. and Nimbvikar et al., found that antiphospholipid antibodies are not absent in primary systemic vasculitis and that a subset of such patients may meet criteria for APS [9, 10]. This does not prove causality, but it does support the idea that the interface between vasculitis and thrombophilia is biologically plausible and clinically relevant. Recent advances in APS pathophysiology further support this thromboinflammatory interface. Mechanisms such as complement activation, neutrophil extracellular trap formation (NETosis), endothelial microparticle release, and endothelial microRNA dysregulation have all been implicated in APS-associated vascular injury and thrombosis. Interestingly, several of these pathways are also linked to endothelial activation and immune-mediated vascular inflammation observed in systemic vasculitides. Although direct evidence in PAN–APS overlap remains limited, these shared mechanisms provide a plausible biological framework for the coexistence of necrotizing vasculitis and thrombotic vasculopathy within the same vascular territory. In that context, PAN associated with APS may be understood not simply as the accidental coexistence of two rare entities, but as a distinct overlap phenotype in which immune-mediated vascular inflammation and thrombosis interact in a mutually aggravating manner. From this perspective, the PAN–APS association should not necessarily be viewed as a coincidental coexistence of two independent rare disorders, but potentially as a complex thromboinflammatory vascular syndrome with overlapping pathogenic pathways.

The clinical distribution of organ involvement in the reviewed cases also deserves comment. Renal disease was especially prominent. Several reports documented renovascular hypertension, renal artery stenosis or occlusion, renal infarction, and microaneurysms [2, 7, 10]. This pattern is highly suggestive of PAN, but the concomitant presence of antiphospholipid antibodies raises the possibility that thrombosis worsens renal ischemia and contributes to loss of function. Neurologic involvement was likewise notable, ranging from ischemic stroke to catastrophic cerebral thrombosis [3, 4, 8, 10]. Morelli et al. also suggested that APS-related valvular heart disease may have contributed an embolic source in their patient, illustrating that neurologic injury in the overlap syndrome may be multifactorial rather than purely vasculitic [3]. Peripheral ischemia was another recurring and particularly severe feature, sometimes culminating in digital necrosis or major amputation [6, 9, 10]. Together, these observations suggest that PAN–APS should be suspected especially in patients with medium-vessel vasculitic features accompanied by disproportionately severe ischemic or thrombotic events.

The therapeutic implications of this overlap are substantial. Treatment of severe PAN typically relies on high-dose glucocorticoids and, in organ-threatening disease, cyclophosphamide [10, 11]. APS, however, requires antithrombotic treatment, usually long-term anticoagulation in definite thrombotic disease [12]. In PAN–APS overlap, these strategies may be simultaneously necessary, but they also generate tension. Dasgupta et al. made this dilemma explicit: because of widespread aneurysms, the team initially deferred anticoagulation due to bleeding risk, but an iliofemoral thrombosis later forced warfarin therapy [2]. This concern is clinically important, as anticoagulation in the setting of visceral aneurysms may increase the risk of life-threatening hemorrhage, whereas failure to anticoagulate a true APS-driven thrombotic process may permit recurrent infarction or limb loss [2, 6, 9, 10]. In other words, the management of these patients is not simply additive; it requires continuous reassessment of whether the dominant process is inflammatory, thrombotic, or both. This therapeutic dilemma remains unresolved due to the absence of evidence-based recommendations specifically addressing PAN–APS overlap syndrome. Consequently, individualized management based on vascular imaging, thrombotic burden, aneurysm distribution, and disease activity becomes essential.

Outcomes in the published cases reinforce the seriousness of this association. Some patients improved with immunosuppression alone or with combined immunosuppressive and anticoagulant therapy [1, 2, 7, 9, 10], but others had catastrophic courses despite apparently appropriate treatment [4, 6]. Han et al. reported a devastating case of PAN complicated by catastrophic APS, ending in death after cerebral infarction [4]. Musuruana and Cavallasca described progressive ischemic complications leading to bilateral limb loss and subsequent fatal myocardial infarction despite immunosuppressive and anticoagulant treatment [6]. These reports indicate that PAN–APS overlap may be associated with higher morbidity than isolated PAN and may justify closer monitoring and earlier therapeutic escalation in selected patients.

The recent report by Nimbvikar et al. adds a modern and particularly valuable perspective by emphasizing that no validated diagnostic tool currently exists to distinguish inflammatory vasculitis from thrombotic vasculopathy when both are plausible [10]. Their discussion points toward a practical future agenda: integrated evaluation using clinical features, antibody profile, tissue histology, angiographic pattern, and possibly newer imaging modalities. Although this remains speculative, the concept is persuasive. A differentiation framework that weights constitutional symptoms, neuropathy, myalgias, biopsy-proven medium-vessel inflammation, angiographic microaneurysms, persistent antiphospholipid antibodies, and objective thrombosis could be useful both clinically and in future observational studies. For now, however, diagnosis remains fundamentally clinicopathologic and heavily dependent on physician judgment.

This review has limitations that should be acknowledged. The evidence base is composed almost entirely of isolated observations, with inevitable publication bias favoring severe or unusual presentations. This limitation reflects the extreme rarity of the PAN–APS overlap syndrome rather than deficiencies in the systematic methodology itself. In rare diseases, systematic reviews frequently rely on case-based evidence, which nevertheless remains valuable for recognizing clinical patterns, diagnostic challenges, and therapeutic implications. The diagnostic criteria applied across decades were not uniform, and some early cases predated current APS classification criteria [12]. In addition, not all reports provided complete histopathologic confirmation of both processes, which means that some cases may represent true coexistence whereas others may reflect mimicry or sequential predominance of one disorder over the other. Nevertheless, despite these limitations, the consistency of the clinical message across reports is striking: PAN and APS can coexist, the overlap may be severe, and failure to recognize the contribution of both inflammation and thrombosis may lead to suboptimal treatment.

In summary, the available literature supports the interpretation that PAN associated with APS is a rare overlap syndrome characterized by combined necrotizing vasculitis and thrombotic vasculopathy. The most useful clues appear to be the coexistence of classic PAN features such as visceral microaneurysms, medium-vessel biopsy findings, neuropathy, and systemic inflammation with persistent antiphospholipid antibodies and objectively documented thrombotic events. From a practical standpoint, clinicians should consider this overlap whenever ischemic manifestations seem disproportionate, recurrent, or insufficiently explained by vasculitis alone. Earlier recognition may help optimize the balance between immunosuppression and anticoagulation, which remains the core therapeutic challenge in this setting [2, 6, 9, 10].

## Data Availability

Not applicable. This study is based on previously published literature.
